# Supplementary material for: Involvement in Fertilization and Expression of Gamete Ubiquitin-Activating Enzymes UBA1 and UBA6 in the Ascidian Halocynthia roretzi
Source: Int J Mol Sci. 2023 Jun 26;24(13):10662. doi: 10.3390/ijms241310662 (PMC10341865; doi:10.3390/ijms241310662)
Supplement: Supplementary file 1 [file ijms-24-10662-s001.zip › ijms-2439318-supplementary.pdf]

Supplemental data

Table S1. ELISA of antiserum and the purified antibody raised against HrUBA1

Dilution (-times)	Preimmune Serum*	UBA1 Antiserum*	UBA1 antibody** affinity-purified
		A_{450}	
1,000	0.042	> 2.000	> 2.000
2,000	0.038	> 2.000	> 2.000
4,000	0.035	> 2.000	> 2.000
8,000	0.034	> 2.000	> 2.000
16,000	0.035	1.769	> 2.000
32,000	0.038	1.407	> 2.000

* Mean value of two experiments.
** Concentration of the purified antibody: 2.2 mg/ml.

Table S2 ELISA of antiserum and the purified antibody raised against HrUBA6

Dilution (-times)	Preimmune Serum*	HrUBA6 Antiserum*	UBA6 antibody** affinity-purified
		A_{450}	
1,000	0.040	1.995	> 2.000
2,000	0.044	1.277	> 2.000
4,000	0.036	0.867	1.627
8,000	0.035	0.550	1.231
16,000	0.035	0.374	0.933
32,000	0.039	0.249	0.865

* Mean value of two experiments.
** Concentration of the purified antibody: 1.2 mg/ml.

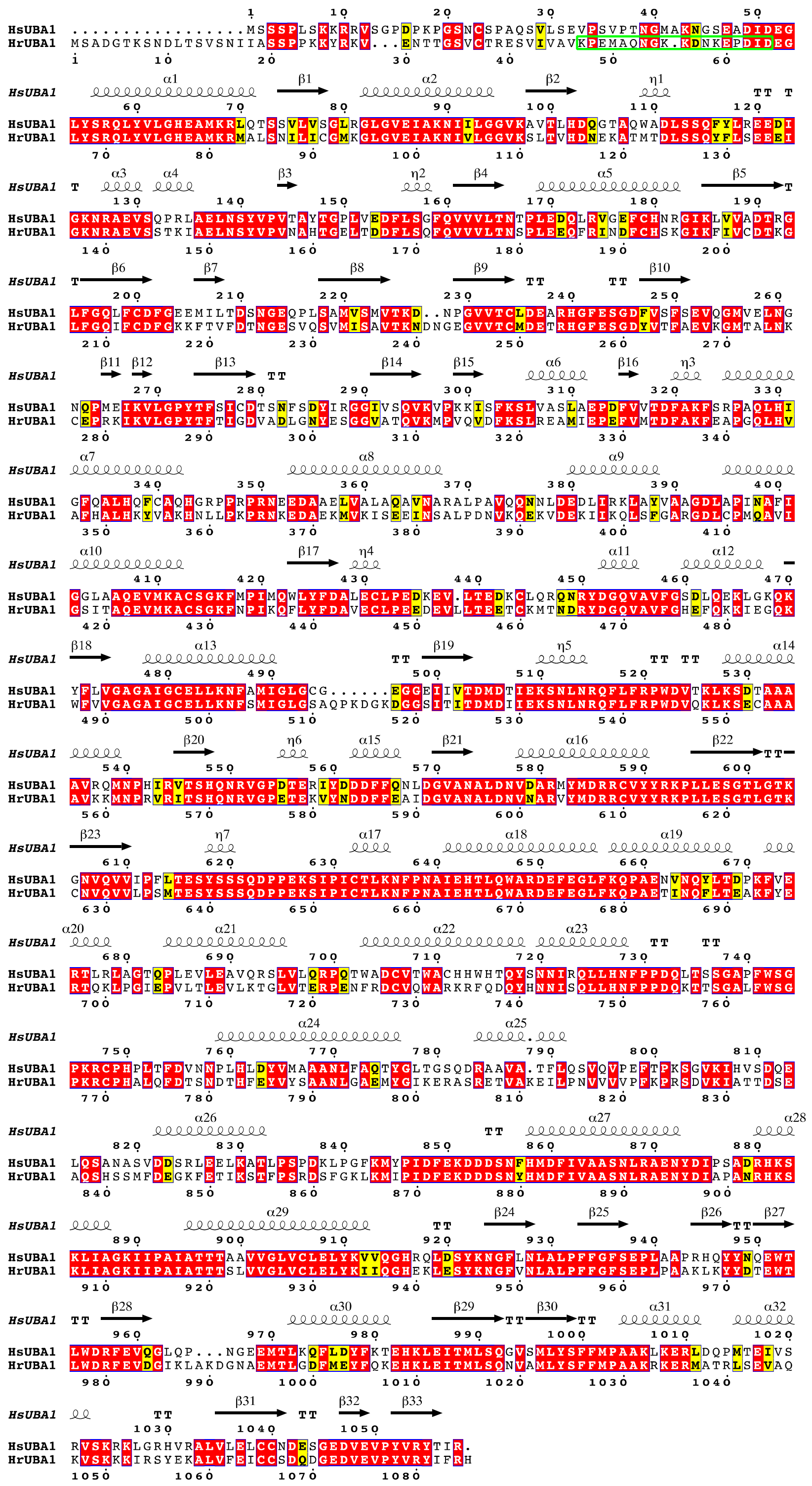

Figure S1. Sequence alignment of human and ascidian UBA1.

Sequence alignment of full-length HsUBA1 and HrUBA1 was performed with Clustal Omega and displayed using ESPript 3.0 [42]. The secondary structure is represented with arrows (B sheets) and helices (a- and **η**-helices) that are provided by PDB code 6DC6 [29]. Lys47 to Asp64 in HrUBA1 depicted by a green box was used for an antigenic peptide.

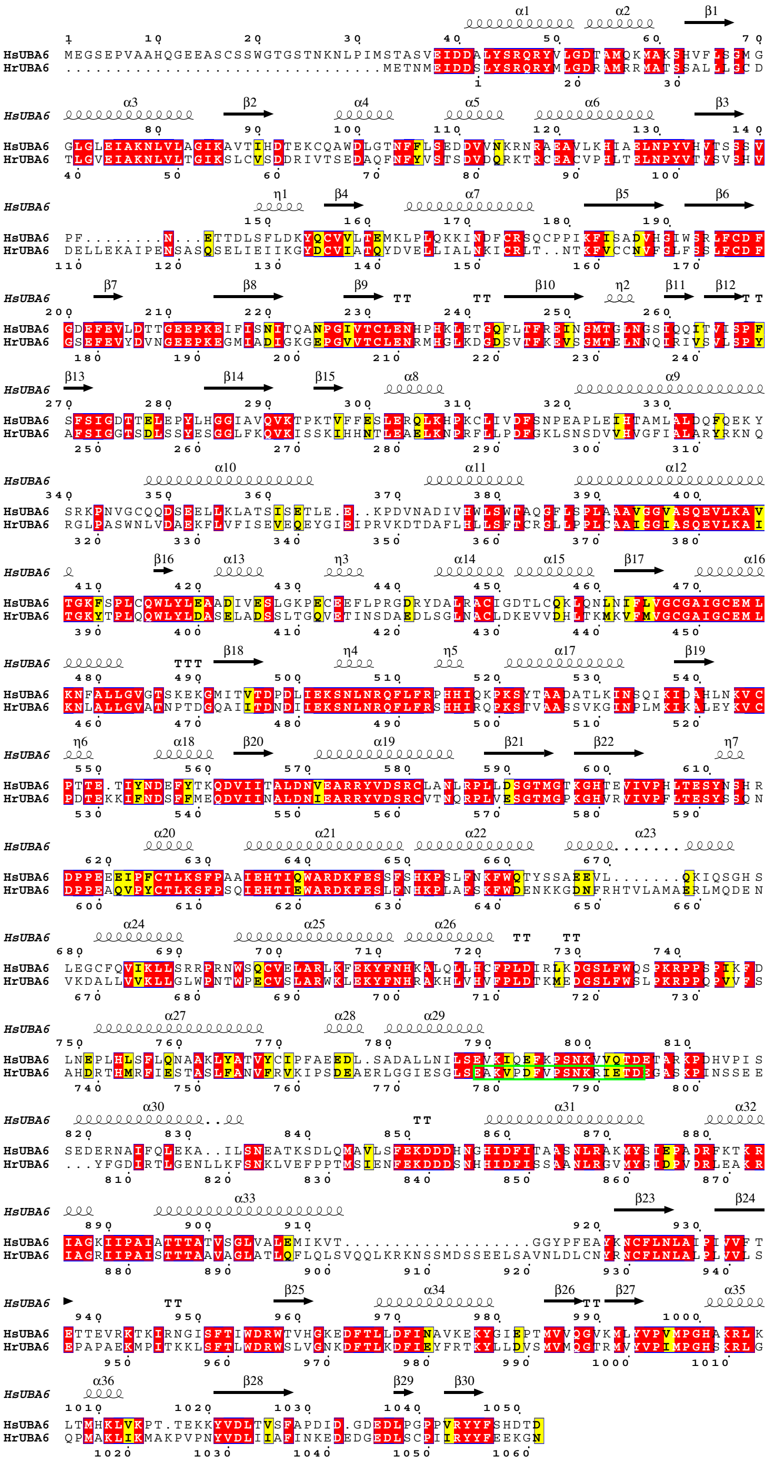

Figure S2. Sequence alignment of human and ascidian UBA6.
Sequence alignment of full-length HsUBA6 and HrUBA6 was performed with Clustal Omega and displayed using ESPript 3.0 [42]. The secondary structure is represented with arrows (B sheets) and helices (a- and **η**-helices) that are provided by PDB code 7PVN [29]. Glu778 to Asp794 in HrUBA6 depicted by a green box was used for an antigenic peptide.

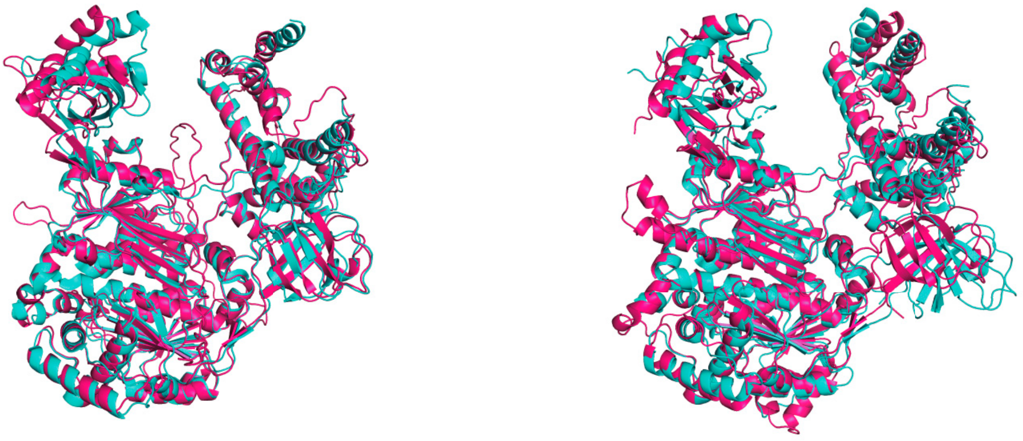

HrUBA1&HsUBA1 HrUBA6&HsUBA6

Figure S3. Comparison of human and ascidian UBA1 and UBA6.
Ascidian UBAs, HrUBA1 (left) and HrUBA6 (right), are colored pink, and human UBAs, HsUBA1 (left) and HsUBA6 (right), are colored cyan.

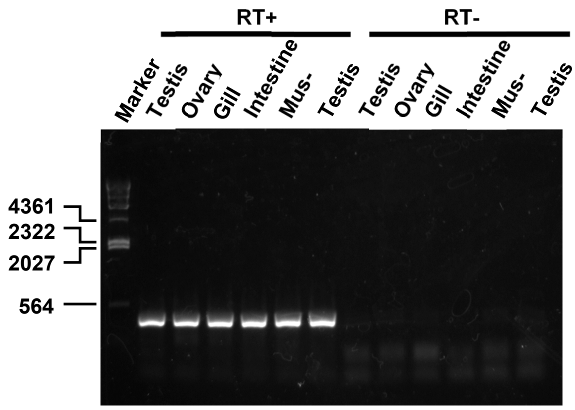

Figure S4. PCR products of the EF-1α gene using cDNAs from various tissues or organs.
Before real-time PCR, cDNA was prepared from the testis, ovary, gill, intestine, and muscles, and the quality of cDNA was tested by PCR using *EF-1α* primers. A single band, whose estimated size is 363 bp, was detected only after reverse transcription, indicating that there is no appreciable contamination of genomic DNA.
